# Generation, Transfer, and Loss of Alternative Oxidase Paralogues in the *Aspergillaceae* Family

**DOI:** 10.3390/jof9121195

**Published:** 2023-12-14

**Authors:** Michel Flipphi, Alexandra Márton, Vivien Bíró, Norbert Ág, Erzsébet Sándor, Erzsébet Fekete, Levente Karaffa

**Affiliations:** 1 Department of Biochemical Engineering, Faculty of Science and Technology, University of Debrecen, H-4032 Debrecen, Hungary; drir.michelflipphi@gmail.com (M.F.); martonszandi7@gmail.com (A.M.); biro0717@gmail.com (V.B.); agnorbi@gmail.com (N.Á.); levente.karaffa@science.unideb.hu (L.K.); 2 Juhász-Nagy Pál Doctoral School of Biology and Environmental Sciences, University of Debrecen, H-4032 Debrecen, Hungary; 3 Institute of Food Science, Faculty of Agricultural and Food Science and Environmental Management, University of Debrecen, H-4032 Debrecen, Hungary; karaffa@agr.unideb.hu

**Keywords:** *Aspergillus*, *Penicillium*, alternative oxidase, *aox* gene multiplicity, gene duplication, paralogous genes, gene loss

## Abstract

Alternative oxidase (Aox) is a terminal oxidase operating in branched electron transport. The activity correlates positively with overflow metabolisms in certain *Aspergilli*, converting intracellular glucose by the shortest possible path into organic acids, like citrate or itaconate. Aox is nearly ubiquitous in fungi, but *aox* gene multiplicity is rare. Nevertheless, within the family of the *Aspergillaceae* and among its various species of industrial relevance—*Aspergillus niger*, *A. oryzae*, *A. terreus*, *Penicillium rubens*—paralogous *aox* genes coexist. Paralogous genes generally arise from duplication and are inherited vertically. Here, we provide evidence of four independent duplication events along the lineage that resulted in *aox* paralogues (*aoxB*) in contemporary *Aspergillus* and *Penicillium* taxa. In some species, three *aox* genes are co-expressed. The origin of the *A. niger* paralogue is different than that of the *A. terreus* paralogue, but all paralogous clades ultimately arise from ubiquitous *aoxA* parent genes. We found different patterns of uncorrelated gene losses reflected in the *Aspergillus* pedigree, albeit the original *aoxA* orthologues persist everywhere and are never replaced. The loss of acquired paralogues co-determines the contemporary *aox* gene content of individual species. In *Aspergillus calidoustus*, the two more ancient paralogues have, in effect, been replaced by two *aoxB* genes of distinct origins.

## 1. Introduction

Cyanide-resistant terminal oxidase (alternative oxidase, Aox; ubiquinol:oxygen oxidoreductase, non-electrogenic; EC 1.10.3.11) has been reported in many organisms, especially in higher plants and fungi [1,2,3,4,5]. Aox is a mitochondrial inner membrane enzyme encoded in the nuclear genome and an ‘alternative’ to electron flow via the cytochrome-dependent respiratory pathway [3]. The site of the branching point from the main chain is at the level of Coenzyme-Q. Therefore, the alternative path is resistant to the inhibitors of Complex III and IV, such as cyanide, nitric oxide or azide, but can be blocked selectively by aromatic hydroxamic acids like salicylic-hydroxamate (SHAM [6]). Compared to the cytochrome pathway, the Aox pathway moves fewer protons across the inner mitochondrial membrane to generate a proton motive force to be used to synthesize ATP, as Complexes III and IV of the mitochondrial electron transport system are bypassed, and Aox lacks proton pumping activity. As a consequence, this pathway provides only 40% of the normal levels of ATP for energy conservation via oxidative phosphorylation.

Aox is induced by stresses such as wounding, chilling, drought, osmotic stress and pathogen attack, in addition to treatment with salicylic acid, hydrogen peroxide or with inhibitors of the cytochrome-dependent respiratory chain. During fungal overflow metabolism in fungi, such as citric acid fermentation by *Aspergillus niger*, Aox uncouples the re-oxidization of NADH from ATP synthesis, thereby allowing carbon catabolism to continue even when mycelia do not require high energy levels [7,8]. The recycling of reducing equivalents (NAD^+^) for primary catabolism is the predicted role of Aox in *Microsporidia*, a phylum of obligate endoparasitic fungi that have no mitochondrial DNA and, hence, no cytochrome electron transport chain [9]. In *Cryptomycota*, Complex I of the respiratory chain is absent, and the alternative respiratory pathway is composed of alternative (type-2) NADH dehydrogenases, and Aox operates to recycle NAD^+^ without any protons translocated [10].

Alternative oxidase is nearly ubiquitous in fungal phyla with representative genome-sequenced. There are some ascomycete taxa lacking the alternative mitochondrial terminal oxidase, including the family of *Saccharomycetideae* and the taxa of unicellular *Taphrinomycotina*. Fungal Aox has sparsely been studied as; it is absent from the fungal “model organisms” of *Saccharomyces cerevisiae* and *Schizosaccharomyces pombe*.

On the other hand, *aox* gene multiplicity appears to be rare in fungi. Three occasions of concurring paralogues have been documented. In the yeast *Candida albicans* (*Debaryomycetideae* family, *Saccharomycotina* subphylum), two differentially expressed, neighbouring genes are orientated in tandem [11]. Furthermore, sequence-related but genetically unlinked genes called *aod-1* and *aod-3* have been described in *Neurospora crassa* [12]. We recently described a third instance, where a rare second *aox* paralogue named *aoxB* (the primary enzyme AoxA encoded by the ubiquitous *aoxA* gene) was found scattered in four species of *Aspergillaceae* amongst hundreds of genome-sequenced member species [13]. Importantly, in the *Aspergillus niger sensu stricto* complex—where more than 80 genome sequences are available for comparison—we identified five mutations in this acquired *aoxB* gene that could be used to distinguish six taxa in this species complex known to be notoriously difficult to subdivide [13]. 

An indication that this rare *aoxB* paralogue may have been transferred horizontally is the presence of a divergently transcribed, equally rare paralogue gene for an alternative (type-2) NADH dehydrogenase (non-electrogenic), called *andB*, which is exclusive to the same four species. To broaden these efforts, a more detailed investigation into the origins of *aox* paralogues in species of *Aspergillus* and *Penicillium* was undertaken in this paper. We will show that paralogous *aox* genes have been generated at least four times during the evolution of *Aspergillaceae*.

## 2. Materials and Methods

### 2.1. Mining of Alternative Oxidase Genes, Intron–Exon Structure Conservation, and Gene Synteny

The coding sequences of alternative oxidase genes (ATG—stop codon) were mined upon TBLASTN screening of the DNA databases on the National Center for Biotechnology Information (NCBI) servers, primarily the Whole-Genome Shotgun contigs (WGS) database, using the available online tools [14]. Using the AoxA protein from *Aspergillus tubingensis* strain WU-2223L (Previously known as *Aspergillus niger* WU-2223L) [15,16] as the query, sequence-similar alternative oxidase genes were searched for, apparently encoding peptides with 55–65% amino acid identity to the query protein, in particular, with the enzyme domains encoded in exons 2 and 3. The N-terminal mitochondrial signal and membrane anchor (helix domain) are far less sequence conserved between Aox paralogues coexisting in the same fungus. TBLASTN screens were run with near default settings, although the Expect Threshold stringency was lowered to 1000, and the Gap Cost reduced to Existence 10; Extension 1, while composition adjustment was omitted and low complexity regions were not filtered. For a few fungi, the genome sequences are located in the Refseq genome database. We did not use the results of automated annotation at NCBI (“Models” or “mRNA”) nor protein databases. To generate estimations of sequence similarity (% of amino acid or DNA identity), we ran Clustal Omega multiple sequence alignments [17] that produce percent identity matrices as part of the output.

We included *Aspergillus* species exclusively available from the Mycocosm depository of fungal genome sequences of the US Dept. of Energy (DOE) Joint Genome Institute (JGI; https://mycocosm.jgi.doe.gov/mycocosm/home, accessed on 23 April 2023) [18], initially for the lack of genome sequences of section *Usti* species in the NCBI databases. We obtained permission to use JGI-lodged genome assemblies generated by the *Aspergillus* whole-genus sequencing project (Principal Investigator: Dr. Scott Baker at the US Dept. of Energy Joint Bioenergy Institute) to identify any paralogous *aox* genes in *Aspergilli*. 

To corroborate orthology and paralogy among sequence-similar genes, TBLASTN hits and their local environment were inspected for intron positional conservation and for the local synteny around the *aox* genes. With few exceptions, all *aox* genes in the taxonomic order of the *Eurotiales*—*aox*A orthologues and paralogues alike—have two phase-two introns bounding a central exon with a length of about 300 nt, usually 291 nt. The genome browsers of the respective JGI genomes of species with publicly available genomes (i.e., those highlighted in light green) were used to inspect the direct environments of the paralogous *aox* loci. Here, we use the JGI-based annotation to determine the orientation of the neighbouring genes and their predicted function to achieve indications in terms of possible gene synteny.

### 2.2. Maximum-Likelihood Phylogenetic Analysis

*Eurotiales*, *Onygenales* and *Lecanoromycetes* Aox proteins were first aligned using Multiple Sequence Alignment with Fast Fourier Transform (MAFFT, version 7) [19,20] using E-INS-i iterative refinement and the BLOSUM45 scoring matrix (fixed variables). The resulting multiple sequence alignments (MSAs) were subsequently trimmed using BMGE (Block Mapping and Gathering using Entropy: [21]) to condense and optimize the ensemble of the highly informative regions, utilizing the substitution matrix BLOSUM55 and a block size of 4 (fixed settings). BMGE-trimmed alignments were then used to infer Maximum Likelihood (ML) trees with PhyML (version 3; online module) [22] employing the general replacement matrix LG [23] with the following settings: invariable sites, estimated; substitution rate, gamma; number of substitutions, 4; gamma-shape, estimated. ML trees were drawn with FigTree version 1.4.3 and rooted in the designated outgroup of *Lecanoromycetes* AoxA: the outgroup was subsequently eliminated via subtree selection. Branch stability was assessed with approximate Likelihood Ratio Tests (aLRTs) [24] integral to PhyML operation using default settings.

The presence of paralogues in phylogenetic analyses often leads to distortion of the underlying topology of orthologue sequences. We studied each of the *aox* multiplication events individually to obtain an indication of the origin of that particular *aox* gene duplication, notwithstanding the existence of other paralogues. Considerable topological instability becomes apparent after the addition of Aox paralogues into mixed phylogenies with the AoxA proteins, mostly affecting the interrelations amongst the *Aspergillus* sections. By contrast, paralogous Aox clades themselves appear monophyletic. 

### 2.3. Confirmation of Expression with Extant RNA Sequence Reads

We identified perfectly matching RNA sequence reads confirming intron excision via BLASTN screening of the species-designated Sequence Read Archives (SRAs) deposited at NCBI. We did not need to create SRA resources ourselves to confirm the gene models and transcript splicing of the *aox* genes identified in this work. We employed 60 nt-long sequences covering the exon fusion site produced by the predicted intron excision as query material. Imperfect SRA reads (<98% identity) were generally ignored as evidence of splicing.

### 2.4. Expression Verification of aox Paralogous Genes

The fungi used to typify the various independent *aox* duplication events reported in this work, giving rise to extant *aox* paralogues (i.e., *aoxB1*; *aoxB2-1*; *aoxB2-2*; *aoxB3*; *aoxB4*) in species of the sister genera *Aspergillus* and *Penicillium* are listed in Table 1, along with their original sources and the accession numbers of their determined cDNA sequences. The list includes a specimen of the *Trichoderma asperellum*/*asperelloides* taxon participating in the lateral transfer of an *Aspergillus*-born *aox* gene (see Results and Discussion section). The rare *aoxB* gene found intact in one clade of the *A. niger sensu stricto* complex (typified by strains ATCC 1015 and CBS 147482), as well as in *A. calidoustus*, *A. implicatus* and *Penicillium swiecickii*, was previously identified as part of a gene couple with an equally rare type-2 NADH dehydrogenase paralogue gene (*andB*), unique to these four divergent taxa [13]. In the gene nomenclature adopted to describe the multiple *aox* gene duplication events in the lineage of the *Aspergillaceae*, this previously described paralogue is dubbed “*aoxB1*”. cDNA analysis proves the excision of introns from pre-mRNA, hence, the expression of the studied gene.

### 2.5. Isolation of Total RNA for cDNA Sequence Analyses

Total RNA for first strand cDNA synthesis was isolated from the biomass of submerged cultures. Fungal biomass was generated in 500 mL Erlenmeyer flasks (VWR International Kft., Debrecen, Hungary) containing 100 mL of a synthetic growth medium in a rotary shaker (Infors AG, Basel, Switzerland) at 200–250 revolutions per minute (rpm) and 28–37 °C for 24–48 h, dependent on the fungus. *Trichoderma asperellum*, *A. wentii* and *A. oryzae* were grown on PDB (potato dextrose broth); the medium for *A. oryzae* additionally contained 1% Triton X-100. For *Penicillium rubens* and *A. calidoustus*, a minimal medium was used consisting of 2 g/L KH_2_PO_4_; 8 g/L Na_2_HPO_4_; 0.25 g/L MgSO_4_; 10 g/L (NH_4_)_2_SO_4_; trace element solution (0.1 g/L CaCl_2_; 1 mg/L CoCl_2_; 8.8 mg/L ZnSO_4_·7 H_2_O; 0.39 mg/L CuSO_4_·5 H_2_O; 0.1 mg/L NiSO_4_; 0.08 mg/L Na₂[B₄O₅(OH)₄]·8 H₂O; 0.072 mg/L MnCl_2_; 0.037 mg/L Na_2_MoO_4_; FeSO_4_ 0.1 mg/L) with 1% D-glucose. *A. terreus* culture medium contained 60 mM sodium-acetate as the carbon source, 0.1 g/L KH_2_PO_4_, 3 g/L NH_4_NO_3_, 1 g/L MgSO_4_·7 H_2_O; 5 g/L CaCl_2_·2 H_2_O; 1.67 mg/L FeCl_3_·6 H_2_O, 8 mg/L ZnSO_4_·7 H_2_O and 15 mg/L CuSO_4_·5 H_2_O. *A. sydowii* was grown on PDB in the presence of 2 M NaCl and 2 M MgCl_2_.

Liquid cultures were inoculated with freshly prepared, high-density conidiospore suspensions in a 0.01% Tween-20 solution. Mycelia for RNA isolation were harvested from three independent liquid cultures (i.e., three independent biological replicates) via filtration over Miracloth (Millipore, Merck KGaA, Darmstadt, Germany), washed with distilled water and deep frozen in liquid nitrogen for further processing. Total RNA was isolated from powdered deep frozen biomass using the RNA Plant kit (Macherey–Nagel GmbH & Co., KG, Düren, Germany). Genomic DNA (gDNA) was isolated from the same biomass using the Macherey–Nagel NucleoSpin Plant II kit (Macherey–Nagel GmbH & Co., KG, Düren, Germany).

### 2.6. Polymerase Chain Reaction (PCR) and cDNA Sequence Determination

First-strand cDNA was synthesized from total RNA with an Oligo(dT) primer using the RevertAid First Strand cDNA Synthesis Kit (Thermo Scientific, Thermo Fisher Scientific, Waltham, MA, USA). The produce from first-strand cDNA synthesis was then used as the template for PCR reaction(s). PCRs were performed with gene-specific oligonucleotide primer pairs (Supplementary Table S1; Integrated DNA Technologies, Leuven, Belgium) and DreamTaq DNA Polymerase (Thermo Scientific, Thermo Fisher Scientific, Waltham, MA, USA) in a T100TM Thermal Cycler (Bio-Rad, Bio-Rad Hungary Ltd., Budapest, Hungary). The designed primer pairs were verified for their performance on the gDNA template. The cycling conditions after initial denaturation at 95 °C (3 min) were: 35 cycles of 95 °C for 30 s, 54 °C for 1 min, and 72 °C for 30 s/min, followed by one post-cyclic elongation at 72 °C (5 min). Purified PCR fragments (NucleoSpin Gel & PCR Clean-up, Macherey-Nagel GmbH & Co., KG, Düren, Germany) were cloned into the bacterial vector pGEM-T Easy (pGEM-T Easy Vector System I, Promega Corporation, Madison, WI, USA). Plasmid DNA was isolated using the NucleoSpin Plasmid EasyPure kit (Macherey-Nagel GmbH & Co., KG, Düren, Germany). Plasmid DNA from three independent clones (three technical replicates) was sequenced over both strands using universal primers hybridizing to the vector (Eurofins Genomics, Ebersberg, Germany). The open reading frame of the determined cDNA sequences (i.e., ATG to stop codon) were deposited at GenBank (see Table 1 for the respective accession numbers).

## 3. Results and Discussion

### 3.1. aox Paralogous Genes Have Been Generated Independently at Four Different Occasions in the Aspergillaceae

A survey of the whole fungal kingdom suggested that *aox* gene multiplicity—generally a rather rare condition—occurs multiple times in the *Eurotiales* order (*Eurotiomycetes* class, *Pezizomycotina* subphylum), particularly in the *Aspergillaceae* family. Preliminary phylogenetic analysis implied that most *aox* paralogous genes in *Aspergillaceae* have their origin within the larger clade of the *Eurotiomycetidae* subclass constituting the sister orders *Eurotiales* and *Onygenales*. We omitted one mixed group of *Eurotiales* secondary Aox proteins from *Rasamsonia emersonii*, *Evansstolkia leycettana*, *Monascus*, *Aspergillus clavatus*, *A. cejpii* and *A. thermomutatus*, that consistently clustered deep in the *Dothideomycetes* AoxA clade (i.e., another taxonomic class) from further analysis. 

**Figure 1:**
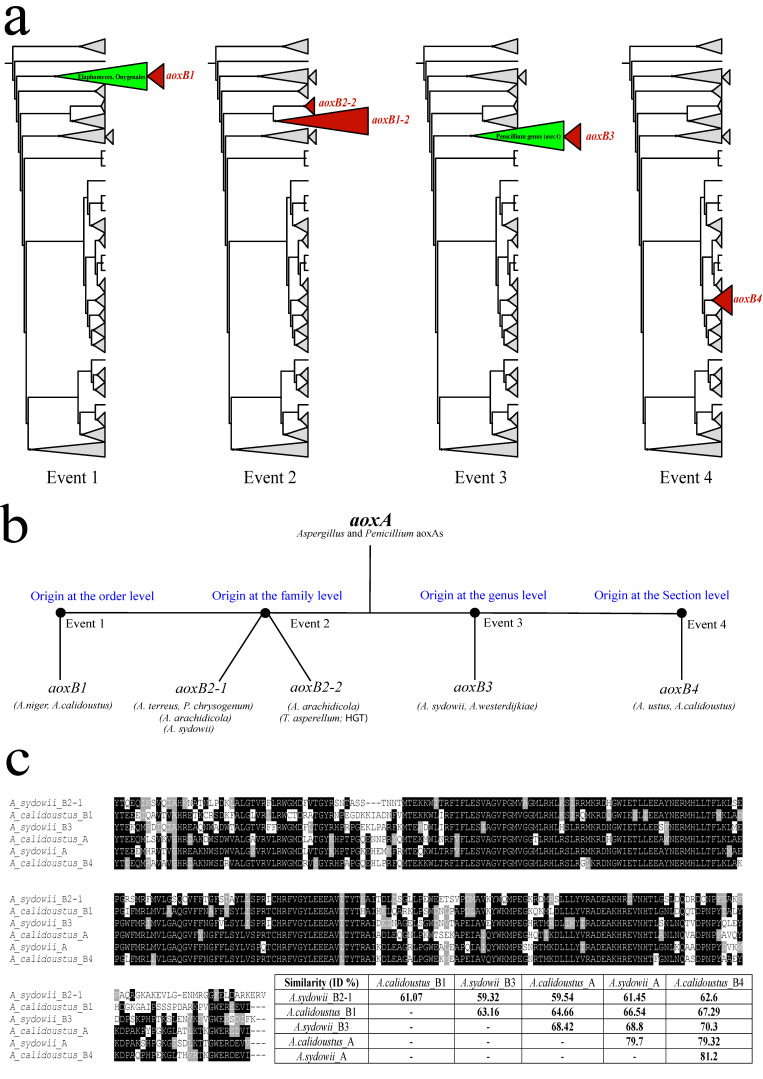
Overview of the independent origins of alternative oxidase paralogous genes in the *Eurotiales* order and the sequence similarity between the individual paralogous Aox’s. (**a**) A section of a ML tree of 531 Aox proteins from 351 species of *Eurotiales*, *Onygenales* and *Lecanoromycetes* was used. All species in the survey have the ubiquitous *aoxA* gene, but some species of *Aspergillus* and *Penicillium* have one or two additional paralogous (*aoxB*) genes named after the duplication event they emerged from (i.e., B1, B2, B3 or B4). The distinction between AoxB2-1 and AoxB2-2 is explained in the Results and Discussion section. The rooted section with only the *Eurotiales-Onygenales* proteins was drawn as a cladogram of collapsed taxa. Clades of related proteins collapsed at the level of Sections for *Aspergillus*, at the genus level for other *Aspergillaceae* or at the family level for the other taxa of *Eurotiales.* In the four schemes, the cladogram is the same, but in each, one of the four events duplications is named and highlighted by the red triangle. (**b**) Graphic summary of the ML analysis (Supplementary Figure S1). The source of each contemporary paralogous *aoxB* pedigree is the ubiquitous *aoxA* gene. Four events of *aox* gene duplication were identified and numbered from 1 to 4. The contemporary paralogue genes emerging therefrom are named accordingly. Some representative species are given for each of the paralogous clades. (**c**) Alignment of the peptides encoded by the three paralogous *aox* genes in *A. calidoustus* (i.e., *aoxA*; *aoxB1*; *aoxB4*) and the three paralogous *aox* genes in *A. sydowii* (i.e., *aoxA*; *aoxB2-1*; *aoxB3*). Aligned peptides start with a conserved tyrosine [Y] coded by the first intact codon in exon 2 of each of the encoding genes, all with fully conserved three-exon gene model. Identical amino acids are shaded on the black background. The amino acid similarity figures (% ID) between each couple of Aox proteins can be extracted from the matrix.

To investigate the origins of *aox* paralogues in species of *Aspergillus* and *Penicillium*, we collected the DNA sequences coding for more than 500 alternative oxidases from about 350 species of *Eurotiomycetidae* and *Lecanoromycetes*, mostly from the freely accessible DNA databases at the NCBI in April 2023. Almost all *Eurotiomycetidae aox* genes we collected typically have two phase-two introns bounding a central exon of about 300 nt, usually 291 nt (exceptions to the conserved gene model are mentioned below in Section 3.2 and Section 3.5). After manual deduction of the intron–exon structures and subsequent translation of the coding sequences after removal of the introns, a maximum likelihood (ML) tree for the updated alternative oxidase complement of 531 proteins in 351 mainly *Eurotiales* species was generated and then rooted with a homogeneous clade of uniquely *Lecanoromycetes* AoxA proteins. From this tree, we deduced that paralogous *aox* genes have been generated (at least) four times during the evolution of the *Aspergillaceae*. Figure 1a graphically shows the four independent paralogous clades (alternating in red) and the approximate sites of their respective connection with the AoxA backbone (351 proteins AoxA). Supplementary Figure S1 shows the full, circular version of the ML tree (531 Aox) without clade collapses, used as the basis of the cladograms in Figure 1. Figure 1b schematically summarizes the relations between the AoxA pedigree and the four independent duplication events of which descendants persist to this present day. Figure 1c highlights the considerable sequence similarity between each of the Aox paralogues for the conserved enzyme domains encoded by the second and third exons. One duplication event appears to have occurred before the separation of the *Aspergillus* and *Penicillium* genera and comprises about a hundred paralogous Aox proteins (see below, Section 3.2). Subsequent gene loss of the acquired paralogues must have taken place frequently in evolution of the extended *Aspergillus* genus as there are whole sections (and series) of *Aspergillus* where only the ubiquitous AoxA (*aoxA* gene) is present. 

We have described one of the four events in our previous paper on the rare *aoxB* paralogue found in the *Aspergillus niger sensu stricto* complex and in three other highly divergent species of *Aspergillaceae* [13]. In the context of this current work, we renamed this rare *aox* paralogue *aoxB1* (Figure 1a, scheme at the left) because its origin is the furthest away from the tips of the terminal branches (i.e., the present), the small, seemingly homogeneous paralogous AoxB1 clade consistently appearing as the sister clade of the *Onygenales* AoxA in alternative ML phylogenies (For clarity, all *Onygenales* species only specify one Aox, AoxA).

### 3.2. Ancient Gene Duplication in an Aspergillaceae Ancestor of Aspergillus and Penicillium

**Figure 2:**
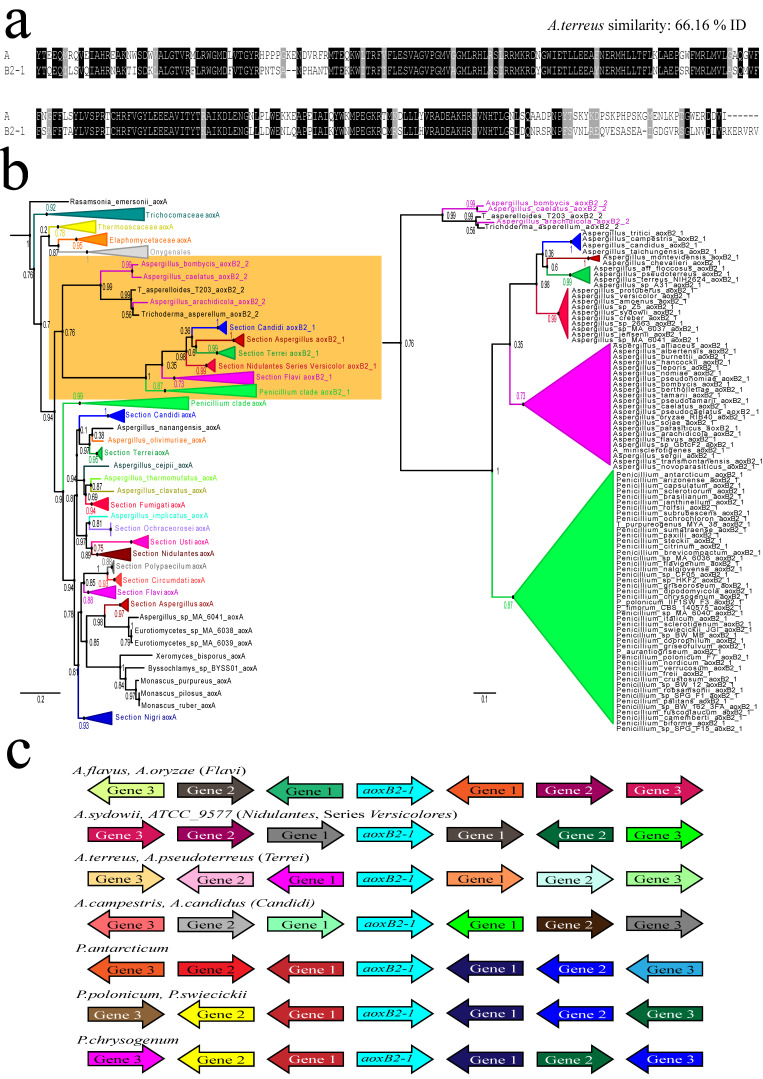
Duplication event 2 and the *aoxB2* paralogous genes emerging from it. (**a**) Amino acid alignment of the conserved C-terminal enzymatic domains of AoxB2-1 and the ubiquitous AoxA proteins from *Aspergillus terreus* (section *Terrei*). Two phase-two introns are position conserved in *Eurotiales* and *Onygenales*, in *aoxA* as well as in the various paralogous *aoxB* genes. Aligned peptides start with a conserved tyrosine [Y] coded by the first intact codon in exon 2. Identical amino acids are shaded in black background; (**b**) relevant mixed maximum likelihood tree for the ubiquitous AoxA proteins and the paralogous AoxB2 proteins. On the left, a summary tree with *Aspergillus* sections, other *Aspergillaceae* genera and other *Eurotiales* families collapsed. The *aoxB2* paralogous clades are boxed in the yellow background. On the right, the paralogous clades are shown in isolation as cartoons of each principal taxon; (**c**) the contemporary environment of the *aoxB2-1* paralogous locus. The orientation and deduced function description of the product of the neighbouring genes were collected from the JGI genome browsers for freely accessible *Aspergillus* and *Penicillium* annotations. The *aox* paralogue gene in the centre is represented by the light blue arrows that always point to the right (5′ to 3′). The different colors represent different predicted functions for the neigbouring gene products. For the sake of clarity, the differently colored arrows are all equal in size and thus do not represent the real size of the coding regions of the neigbouring genes.

Most of the paralogous Aox in the family, including virtually all paralogous *aox* genes present in the *Penicillium* genus, appear to derive from a gene duplication event that must have occurred in early *Aspergillaceae* before the divergence of the sister genera (Figure 2). Most *Penicillium* genomes investigated feature two *aox* genes, although some *Penicillium* taxa (e.g., all species of the series *Roquefortorum*) only have *aoxA*. There were almost 100 paralogues of AoxB2-1 in our collection of 531 proteins. The basal branch of the AoxB2-1 clade is by far the longest in the ML tree, indicative of increased genetic variation. AoxB2-1 has also survived in the majority of species in the *Aspergillus* sections of *Flavi*, *Terrei* and *Candidi* included in the phylogeny (i.e., Supplementary Figure S1) as well as in the series *Versicolores*, one of the taxonomic series defined in the section *Nidulantes* (cf. [32]). Two early divergent section *Flavi* species, *A. avenaceus* and *A. coremiiformis*, do not have AoxB2-1 (at present). Nevertheless, typical for section *Flavi*, the gene encoding the ubiquitous *aoxA* has lost the phase-two intron at the second conserved position. Two out of seven species from our initial collection of 351 species in the early divergent section *Aspergillus*—*A. chevalieri* and *A. montevidendis*—harbour a genuine AoxB2-1 paralogue. In *Aspergillus cristatus* (two genomes public), the paralogue is a recognizable pseudogene, but *A. glaucus* and *A. ruber* only feature *aoxA*. In other sections of *Aspergillus*—*Fumigati*, *Nigri*, *Circumdati*, *Cremei*, and *Usti*, and in the series of section *Nidulantes* other than series *Versicolores*—the duplication AoxB2-1 is absent (at the present). The available species in the series *Unguium*, *Multicolores* and *Nidulantes (*including *A. nidulans*) only harbour the ubiquitous *aoxA* gene and thus must have lost *B2-1*. 

This big paralogue AoxB2-1 clade is linked with a satellite clade of another Aox paralogue, AoxB2-2, which currently persists in only three species of Section *Flavi*—*A. caelatus*, *A. bombycis* (aka *A. luteovirescens*), and *A. arachidicola* (Figure 2b; Supplementary Figure S2). Due to a lack of sequences, it is impossible to determine whether there is direct descendancy or parallel evolution of the two *aoxB2* paralogues, but the three *Flavi* species mentioned above have both AoxB2-1 and AoxB2-2. Omission of the small satellite clade (AoxB2-2) from phylogenies of AoxA-plus-AoxB2-1 resulted in a dramatic shift in the topology of *Aspergillus* AoxA considering the perceived evolutionary relations amongst the *Aspergillus* sections. Closer inspection of section *Flavi* genome data suggested that AoxB2-2 paralogues in three more species, *A. pseudocaelatus*, *A. transmontanensis*, and *A. novoparasiticus*, are heavily degenerated. The six species are not clustered in one and the same clade (cf. [33]) but are distributed over three discernible terminal clades, series *Flavi*, *Kitamyces* and *Nomiarum* (cf. [32]). This unequal distribution implies that other species in these three series have been rid of AoxB2-2, all the while maintaining the AoxB2-1 paralogue. These observations may suggest that the AoxB2-2 paralogue is doomed to disappear.

### 3.3. Lateral Transfer of a Fungal aox Gene between Species of Different Taxonomic Classes

Contemporary paralogous genes are generally the product of gene duplication. The newly acquired copy is passed on vertically to yield defined lineages with the dual presence of paralogues. The absence of the acquired copy from taxa within such taxonomic clades can be explained by gene loss. A rare, alternative path to acquire paralogous genes is horizontal gene transfer (HGT) or lateral transfer. In the current work, we fortuitously identified an unambiguous lateral transfer between long divergent filamentous fungal taxa involving an *aox* gene from an *Aspergillus* section *Flavi* donor and a narrow taxon in *Trichoderma* (*Hypocreaceae* family, *Hypocreales* order, *Sordariomycetes* class; Supplementary Figure S2). Unique to two closely related cryptic species—*Trichoderma asperellum* and *T. asperelloides* [34]—we noticed a second *aox* gene in all 12 genome sequences available at NCBI, of which the protein product consistently and tightly clusters with the evanishing small clade of paralogues AoxB2-2 (described above). The ubiquitous *aoxA* gene in *Trichoderma* species has two phase-zero introns bounding a central exon of 399 nt, an intron–exon structure conserved in all *Hypocreaceae* and in almost all genome-sequenced *Hypocreales.* However, the intron–exon structure of the extra (second) *aox* gene in *T. asperellum*/*asperelloides* includes two phase-two introns binding to a central exon of 297 nt: this is exactly the gene model of the large majority of *aox* genes in the *Aspergillus* and *Penicillium* genera (as well as in other *Eurotiales*; Supplementary Figure S2a). Intron position conservation is diagnostic of orthology (cf. [35]). Clearly, the second *aox* gene is not the product of a duplication in the *Trichoderma aoxA* lineage. The protein most similar to the second Aox in *T. asperellum*/*asperelloides* (345 amino acids) is the AoxB2-2 paralogue of equal length in *Aspergillus arachidicola* (Section *Flavi*, series *Flavi*; Supplementary Figure S2b). These two proteins should be considered orthologues; >86% identical over the complete gene product and >93% identical when the exon-1-encoded amino acids—including the mitochondrial signal peptide—are omitted. Limited gene synteny is conserved upstream of the *aoxB2-2* gene in the opposite participants of the plausible transfer.

### 3.4. A Gene Duplication Seemingly Arising from within the Clade of Penicillium aoxA

**Figure 3:**
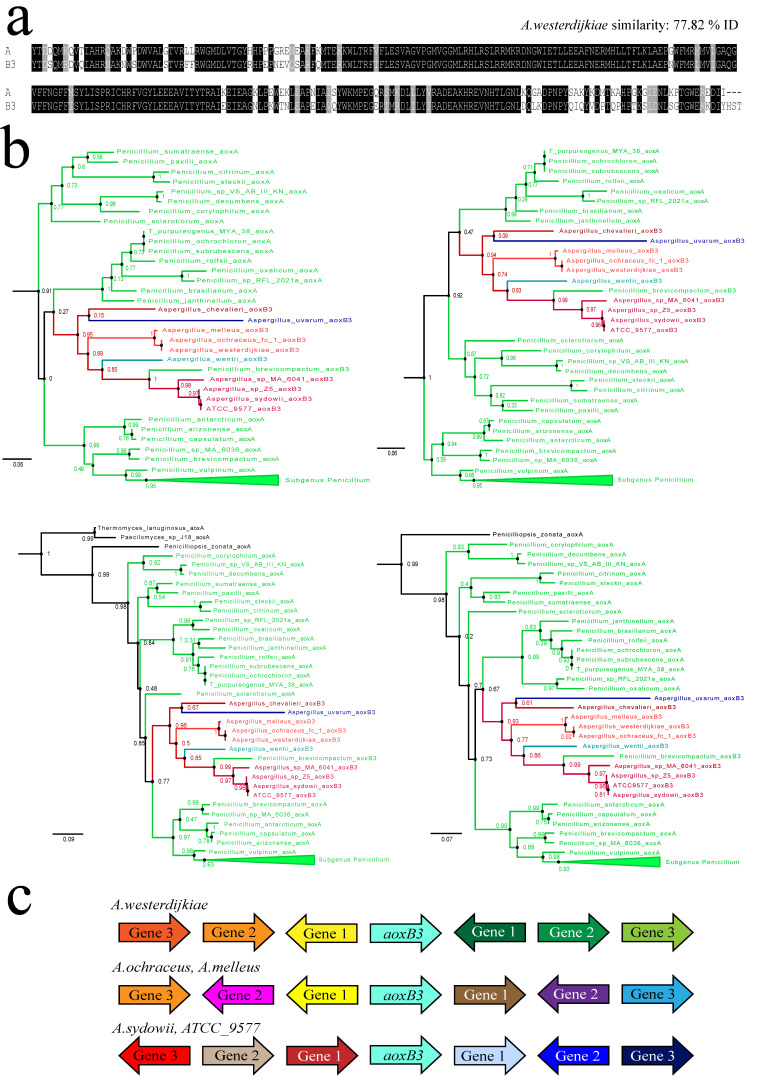
Duplication event 3 and the *aoxB3* paralogous genes emerging from it. (**a**) Amino acid alignment of the conserved C-terminal enzymatic domains of AoxB3 and the ubiquitous AoxA proteins from *Aspergillus westerdijkiae* (section *Circumdati*). Aligned peptides start with a conserved tyrosine [Y] coded by the first intact codon in exon 2. Identical amino acids are shaded in black background; (**b**) Mixed maximum likelihood trees with *Penicillium* AoxA proteins and the paralogous AoxB3 proteins; (**c**) The contemporary environment of the *aoxB3* paralogous locus. The orientation and deduced function of the product of the neighbouring genes were collected from the JGI genome browsers (see legend to Figure 2). The *aox* paralogue gene in the centre is represented by the light green-blue arrows. The different colors represent different predicted functions for the neigbouring gene products.

Another independent event has given rise to a monophyletic clade of *aox* paralogues in ten taxa of *Aspergillus* and one species of *Penicillium*, *P. brevicompactum* (Figure 3). The ten taxa in *Aspergillus* (from the 351 used in Supplementary Figure S1) represent five different taxonomic sections. In section *Circumdati*, three species available at NCBI have the AoxB3 paralogue (Figure 3b), but *A. steynii*, *A. sclerotiorum* and *A. persii* lack it. On the contrary, *Aspergillus uvarum* (subgenus *Circumdati*, section *Nigri*, series *Japonici*) seems to be the only species of section *Nigri* to feature duplication *aoxB3.* A close inspection of the gene sequences identified *A. sydowii* (CBS 593.65) as the principal *Versicolores* that (still) features the AoxB3 paralogue. Gene sequence alignments imply that strains ATCC 9577, AS33 and Z5 co-identify as variants of *A. sydowii* (CBS 593.65). The unassigned strain *Aspergillus* sp. MA 6041 is arguably the only other *Versicolores* available at NCBI with an intact *aoxB3* gene. Interestingly, *A. jensenii*, *A. tennesseensis* and *A. creber* feature a small 3′ terminal remnant of the coding region, implying that the *aoxB3* gene was present but then lost from (these) other *Versicolores*. Due to the lack of available sequences, we observe that AoxB3 occurrence in section *Aspergillus* appears to be restricted to *A. chevalieri* while *Aspergillus wentii* is the only representant of section *Cremei* at NCBI. 

Phylogenetic analyses (Figure 3b) suggest that the *aoxB3* paralogue arose from a *Penicillium* parent rather than from an *Aspergillus* taxon after the separation of the two genera and independent of the earlier *aoxB2-1* duplication. Changing the protein input or the substitution matrix used to build alternative AoxA-plus-AoxB3 phylogenetic trees (with the NCBI-based sequences) does have an influence on the exact “connection” of the monophyletic AoxB3 clade within the *Penicillium* AoxA clade. Figure 3b shows four possible “points of origin” amongst taxa of subgenus *Aspergilloides* or as a sister clade to subgenus *Penicillium* AoxA. The presence of AoxB3 in species of five diverse *Aspergillus* sections—*Cremei*, *Aspergillus*, *Circumdati*, *Nigri*—and in the series *Versicolores* of Section *Nidulantes* remains consistent with vertical inheritance of AoxB3 after acquisition of the paralogue by a common ancestor upon lateral transfer from a *Penicillium* host, followed by numerous independent gene loss events in divergent taxa. Nevertheless, the existence of a third *Penicillium brevicompactum* protein locked within the seemingly monophyletic AoxB3 clade may best be explained by a secondary lateral transfer of the *aoxB3* paralogue from an unknown *Aspergillus* host. Recently, the transfer of a complete gene cluster from a *Penicillium* host to a narrow taxon of *Aspergillus* section *Flavi* species was reported [36].

### 3.5. Recent Gene Duplication at the Basis of Section Usti (Subgenus Nidulantes)

**Figure 4:**
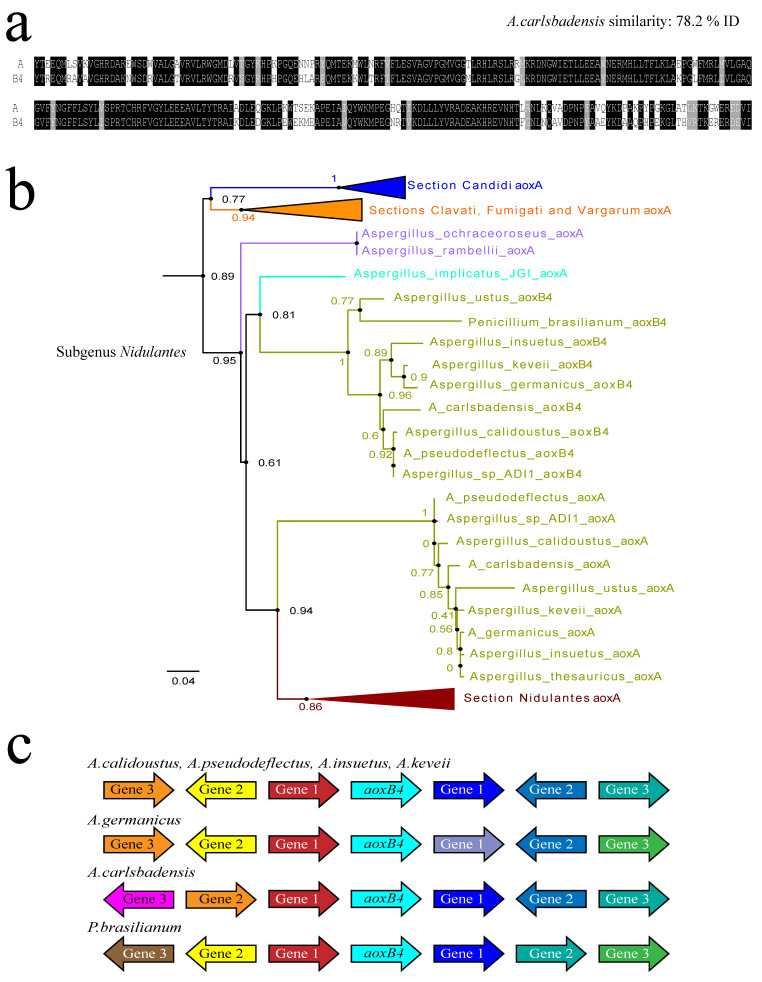
Duplication event 4 and the *aoxB4* paralogous genes emerging from it. (**a**) Amino acid alignment of the conserved C-terminal enzymatic domains of AoxB4 and the ubiquitous AoxA proteins from *Aspergillus carlsbadensis* (section *Usti*). Aligned peptides start with a conserved tyrosine [Y] coded by the first intact codon in exon 2. Identical amino acids are shaded in black background; (**b**) relevant clade of a mixed-input maximum likelihood tree with Subgenus *Nidulantes* AoxA proteins and the paralogous AoxB4 proteins to localize the origin of event 4 in the Subgenus. Clades consisting of sections other than section *Usti* were all collapsed; (**c**) the contemporary environment of the *aoxB4* locus. The orientation and deduced function description of the product of the neighbouring genes were collected from the JGI genome browsers (see legend to Figure 2). The *aox* paralogue gene in the centre is represented by the turquoise arrows. The different colors represent different predicted functions for the neigbouring gene products.

Paralogue AoxB4 is confined to species of the section *Usti* with the illogical exception of the third *aox* gene found in multiple *Penicillium brasilianum* genomes (Figure 4). As there are only two named section *Usti* genomes available at NCBI, we enlarged the sample with the *aox* complement in five closely related species from the Mycocosm whole-genome depository at the US Department of Energy Joint Genome Institute [18] after obtaining permission to use their unpublished genome data to identify their alternative oxidase gene content (see Material and Methods section). The evolutionary relations revealed by alternative AoxA-plus-AoxB4 phylogenies appear to be consistent with a recent gene duplication event near the basis of the section *Usti* (Figure 4b) after the divergence of the section *Ochraceorosei*. This is consistent with the conservation of gene synteny around the locus of *aoxB4* integration (Figure 4c), something that was not observed amongst the present-day produce of the much older duplication events 2 and 3. Remarkably, the original ubiquitous *aoxA* gene has acquired a third intron (phase zero) 55 nt downstream of the 3′ position-conserved phase-two intron: this phase-zero intron is unique to section *Usti aoxA* and does not occur section *Nidulantes*. The most parsimonious explanation for the presence of *aoxB4* in *P. brasilianum* would be a recent lateral transfer from a section *Usti* host after the earlier *aoxB4* gene duplication. From Figure 4c, one can appreciate that the piece of DNA transferred to *P. brasilianum* is considerably bigger than the *aoxB4* gene, comprising at least four neighbouring genes. The amino acid similarity between *Aspergillus ustus aoxB4* and *P. brasilianum aoxB4* is 84.5% identity over the complete width of the protein and almost 90% identity for the peptide product, disregarding exon 1. This last figure is considerably higher than the similarity between the ubiquitous AoxA proteins from *A. ustus* and *P. brasilianum*, ~74% identity (disregarding exon 1).

### 3.6. Verification of the Expression of Alternative Oxidase Paralogous (aoxB) Genes

In our ML phylogenies, no instances of loss of the original *aoxA* gene were observed in the set of 351 species investigated. This suggests that the function of the omnipresent *aoxA* gene cannot be fully replaced by paralogous *aox* genes wherever paralogous *aox* genes coexist. Regardless, a few evolutionary scattered *Aspergillus* and *Penicillium* species have “accumulated” three *aox* genes. We sought to establish whether these paralogue *aoxB* genes are factually expressed or even co-expressed with *aoxA*, or not. Seven species with either two or three *aox* genes in their genome were selected (Table 1): five *Aspergilli*, one *Penicillium* and one *Trichoderma*—the latter species involved in a lateral transfer of a rare *Aspergillus aox* paralogue (see Section 3.3). Paralogue genes originating from each of the duplication events identified (cf. Figure 1) were covered at least once in this set of species. The expression of *aoxA* was also assessed. First, we looked for direct evidence of RNA splicing in extant RNA sequence read archives (SRAs; Supplementary Table S2). *Eurotiales* and *Onygenales aox* genes generally have two position-conserved phase-two introns, bounding a central exon of ~300 nt (usually 291 nt). For *Aspergillus wentii*, we screened computer-assembled RNA contigs covering the predicted exon–exon fusions at the appropriate JGI genome browser for the absence of RNA SRAs in that species. With one exception—the first intron in the *aoxA* transcript in *A. wentii*—we found sequence reads (or EST contigs) for each of the selected *aox* genes, covering the predicted exon–exon fusions within the mRNAs. Thus, all nine *aoxB* paralogues tested were expressed by this criterion. Interestingly, exon–exon fusions covering the predicted introns in both or all three *aox* genes in each species were encountered in the same species-specific SRA database. This implied that the tested paralogue *aoxB* genes were all co-expressed with their original *aoxA* genes (at least in the seven fungi assessed). These include both introns of the laterally transferred, *Aspergillus*-born *aox* gene (i.e., *aoxB2-2*) in *T. asperellum* (see Supplementary Figure S2). We have verified and confirmed the conclusions from the SRA screen by performing targeted RT-PCRs with gene-specific oligonucleotide primers on total RNA samples isolated from fresh liquid cultures of the seven fungi and subsequent sequence analysis of cloned cDNAs (see Materials and Methods section). The complete coding regions in the obtained cDNA sequences were deposited at GenBank (see Table 1 for the accession numbers).

### 3.7. Different Patterns of aoxB Gene Loss in the Aspergillus Genus

The limited representation of *Aspergillus* sections other than *Flavi* and *Nigri* in the public databases (NCBI) resulted in an incomplete view of the inheritance of the *aoxB2-1* and *aoxB3* paralogues. It is plausible to assume that duplication event 2 took place before the divergence of *Aspergillus* and *Penicillium* (Figure 2b) and thus that all emerging lineages must have had the *aoxB2-1* paralogue at their onset (including *Penicillium*). Event 3 must have an independent origin from a *Penicillium aoxA* donor (Figure 3b), explicitly after the separation of the genera. Currently, *aoxB3* is present in taxa belonging to the long divergent subgenera *Cremei* (*A. wentii*), *Aspergillus* (*A. chevalieri*), *Nidulantes* (*A. sydowii*) and *Circumdati* (e.g., *A. westerdijkiae*) strongly suggesting it has its origin in a common ancestor of these subgenera, putatively an ancestor to all current species in the genus. Crucially, no paralogue Aox can apparently fully replace AoxA, which appears omnipresent throughout *Pezizomycotina*. On the other hand, it would appear that duplication *aoxB2-2* is on the edge of extinction in a few now separated narrow taxa in section *Flavi* that explicitly maintain the more sequence-variant *aoxB2-1* paralogue (see Figure 2a). Regardless of its broad occurrence in both subgenera of *Penicillium*, in *Aspergillus* sections *Fumigati*, *Nigri*, *Circumdati*, *Cremei*, and *Usti*, the duplication AoxB2-1 is absent (at the present). The section *Fumigati* stands out for having only the ubiquitous *aoxA* gene, and all transient *aoxB* paralogues from past duplications are lost at present. 

**Figure 5:**
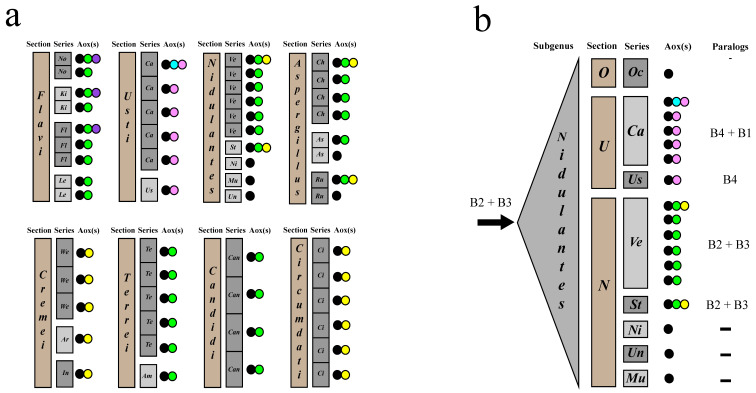
Schematic overview of detectable *aox* gene loss patterns in lineages of *Aspergilli*. (**a**) Occurance and distribution of multiple paralogous *aox* genes in eight *Aspergillus* sections points towards independent gene loss events; (**b**) patterns of *aox* gene loss (B2, B3) and gain (B4, B1: in *Usti*) in sections and series of the subgenus *Nidulantes*. The ubiquitous *aoxA* gene is always present. In both panels, the rectangles at the left of each of the schemes represent the taxonomic lineage, sections and series. The series within each of the sections are given in alternating shades of grey, and their names are abbreviated to a two- or three-letter code; *No*: *Nomiarum*; *Ki*: *Kitamyces*; *Fl*: *Flavi*; *Le*: *Leporum*; *Ca*: *Calidousti*; *Us*: *Usti*; *Ve*: *Versicolores*; *St*: *Stellati*; *Ni*: *Nidulantes*; *Mu*: *Multicolores*; *Un*: *Unguium*; *Ch*: *Chevalierorum*; *As*: *Aspergillus* (series); *Ru*: *Rubri*; *We*: *Wentiorum*; *Ar*: *Arxiorum*; *In*: *Inflati*; *Te*: *Terrei*; *Am*: *Ambigui*; *Can*: *Candidi*; *Ci*: *Circumdati*. To their right, the colored dots represent the presence of maximal three *aox* genes in one species, each of the six *aox* paralogues with its unique color: *aoxA*: black; *aoxB1*: turquoise; *aoxB2-1*: green; *aoxB2-2*: violet; *aoxB3*: yellow; *aoxB4*: magenta. In (**b**), three sections of the *Nidulantes* subgenus are abbreviated with a one-letter code; *O*: *Ochraceorosei*; *U*: *Usti*; *N*: *Nidulantes* (section). In addition, *Oc*: *Ochraceorosei* (series without *aox* paralogous genes not mentioned in (**a**)).

It is likely that independent episodes of paralogue *aoxB* gene loss have taken place in defined pedigrees of *Aspergillus*, eventually resulting in the present-day distribution of these two *aox* paralogues, *aoxB2-1* and *aoxB3*. To track patterns of gene loss of *aox* paralogues in the *Aspergillus* genus more confidently, we expanded our data set with information about *aoxB* genes in the whole-genome sequences deposited at the Mycocosm webpage of the Joint Genome Institute (US Department of Energy) [18]. We gained the necessary permission (see Material and Methods) to use whole-genome data (i.e., DNA contigs) from more than 170 species of *Aspergillus* (situation on 6 September 2023), all part of the *Aspergillus* whole-genus sequencing project (JGI Proposal ID: 1307). Figure 5a summarizes the status of *aox* paralogue genes in eight *Aspergillus* sections with multiple *aox* genes based on this wider set of *Aspergillus* whole-genome sequences. More complete information is found in Supplementary Table S3, deduced from reanalyses, including the extra JGI-lodged genomes. 

Inspection of the extended set showed the presence of *aoxB3* beyond *A. wentii* in the section *Cremei*, with presence in three series of that early divergent section. Likewise, additional species in series *Circumdati* have the *aoxB3* gene, albeit not all species, while other series in this section did not feature *aoxB3* at all (at present). In these diverse sections, the *aoxB3* gene proves to be more persistent than the *aoxB2-1* paralogue. On the contrary, in sections *Flavi, Terrei* and *Candidi*—which like *Circumdati* belong to extended subgenus *Circumdati* (cf. [32])—the *aoxB2-1* paralogue is preserved while *aoxB3* is absent (at present). 

There are also multiple *Aspergillus* taxa that feature both *aoxB2-1* and *aoxB3* at present, although not in all related species grouped in those taxa. This situation concurs with the early divergent section *Aspergillus* and in the series *Versicolores* of the section *Nidulantes*, taxa unambiguously belonging to different subgeneras (Figure 5a). In our initial analysis, we found one named species of each taxon with three *aox* genes, namely *A. chevalieri* and *A.sydowii* (respectively). One can deduce that in the section *Aspergillus*, the series *Aspergillus* and *Rubri* species have lost *aoxB2-1* after the separation from the series *Chevalierorum*. Interestingly, the *aoxB3* paralogue seems more vulnerable to elimination than *aoxB2-1* in *Chevalierorum*; in all available species but *A. chevalieri*, *aoxB3* is lost while *aoxB2-1* persists in all. However, independent gene loss must have occurred recently as one species of series *Rubri* in our extended set—*Aspergillus cumulatus*—features both *aoxB* paralogues, i.e., the original context. 

A complex pattern of *aoxB* gene loss and gain can be observed in the evolution and divergence of the sections and series within the subgenus *Nidulantes* (Figure 5b). Both paralogues arising from events 2 and 3, *aoxB2-1* and *aoxB3*, must have been present in the last common ancestor to the whole subgenus. After the divergences of the *Ochraceorosei* and *Usti*, both paralogues *aoxB2-1* and *aoxB3* have been lost during independent events in both those two sections but were maintained in section *Nidulantes*. In section *Usti*, a fourth duplication event took place, giving rise to the new *aoxB4* paralogous gene. (see also Figure 5a). In addition, *Aspergillus calidoustus*—all six genomes in the databases—trapped a rare copy of the *aoxB1* duplication event (cf. [13]). In *A. calidoustus*, both paralogous *aoxB* genes are thus effectively “replaced” by two other *aoxB* paralogues originating from different duplication events. In the section *Nidulantes*, the species in most constituent series for which genome sequences are available have lost *aoxB2-1* and *aoxB3* on a third occasion (i.e., the fifth and sixth independent gene loss events in the subgenus), including *Aspergillus nidulans*, *A. mulundensis* and *A. unguis*, which all are left with just the ubiquitous *aoxA* gene. Indeed, the series *Stellati* clusters with the series *Nidulantes*, *Multicolores* and *Unguium* (cf. [32]), but *Aspergillus angustatus* has conserved its three aox genes (*aoxA*; *aoxB2-1*; *aoxB3*). Finally, the species in the series *Versicolores* all persist with the *aoxB2-1* paralogue, but most of them have lost *aoxB3* (i.e., the seventh independent gene loss event in the subgenus). The exceptions are *A. sydowii* and *Aspergillus sp*. MA 6041 (see Section 3.2 and Section 3.4), who have both retained *aoxB2-1* and *aoxB3*. Hence, in the two distally related series *Versicolores* and *Chevalierorum*, *aoxB3* seems more transient and *aoxB2-1* more persistent. 

Thus, different patterns of consecutive, independent gene loss events are equally crucial to contemporary *aox* gene content as the original duplication or transfer events, giving rise to *aox* paralogue genes in the extended *Aspergillus* genus.

## 4. Conclusions

Alternative oxidase (Aox) is a non-electrogenic terminal oxidase operating in branched electron transport, oxidizing ubiquinol and reducing molecular oxygen without generating proton motive force over the mitochondrial inner membrane. Aox lowers the energy yield of respiration compared to the canonical electron transport chain and oxidative phosphorylation while dissipating the excess reducing power generated by fast carbon catabolism and moderating oxidative stress via reactive oxygen species, an inevitable byproduct of cytochromic electron transport. In fungi, these activities are positively correlated with sustained overflow metabolism, a feature of immense biotechnological importance. Aox protein is nearly ubiquitous in the fungal kingdom (*aoxA* gene), but *aox* gene multiplicity is rare. Nevertheless, within the *Aspergillaceae* family, and amongst its various industrial cell factories, like *Aspergillus oryzae*, *A. terreus*, *A. niger*, *A. wentii* and *Penicillium rubens*, paralogous *aox* genes coexist. Paralogous genes generally originate from duplication and are inherited vertically. Our study provides evidence for four independent duplication events at different points in evolution that resulted in *aox* (*aoxB*) paralogues in contemporary Aspergilli and Penicillia. The paralogous clades all arise from ubiquitous *aoxA* parent genes but never replace the latter: *aoxA* is actually persistent across filamentous fungi. The most ancient duplication in *Aspergillaceae* must have taken place before the divergence of the genera *Aspergillus* and *Penicillium*. Nevertheless, in some species, three *aox* genes are co-expressed, but there are also whole *Aspergillus* sections and series that must have lost transient *aoxB* content. Different patterns of uncorrelated gene losses were reflected in the *Aspergillus* pedigree, in particular, within the subgenus *Nidulantes*, where we predict seven independent instances of *aoxB* gene loss—involving two different paralogues—in addition to two occasions of *aoxB* gain, the gains involving other *aoxB* paralogues of completely independent origin. Therefore, loss of once-acquired paralogues co-determines the contemporary *aox* gene content within individual fungal species.

## Figures and Tables

**Table 1:** *Aspergillus* and *Penicillium* species used to confirm the expression of paralogous *aoxB* genes originating from the four independent duplication events and the lateral transfer to *Trichoderma asperellum*.

Species	Strain	Relevant Master Accession Numbers	Reference(s) to the Genome Sequences	Source of Live Material	GenBank Accession Numbers [cDNA] * [This Work]
*Aspergillus terreus*	NIH 2624	AAJN [GenBank]	[25]	CBS ***	[aoxA] OR702883 [aoxB2-1] OR702884
*Aspergillus oryzae*	RIB40	JZJM [GenBank]	[26] [25]	CBS	[aoxA] OR683635 [aoxB2-1] OR683636
*Penicillium rubens* **	NRRL 1951	AM920416–64 [EMBL] JAQKAF [GenBank]	[27]	Antibióticos S.A.(León, Spain)	[aoxA] OR702887 [aoxB2-1] OR702888
*Aspergillus wentii*	DTO 134E9	LJSE [GenBank]	[28]	CBS	[aoxA] OR702885 [aoxB3] OR702886
*Aspergillus calidoustus*	SF006504	CDMC [GenBank]	[29]	Hans Knöll Institute (Jena, Germany)	[aoxA] OR714815 [aoxB1] OR631741 [aoxB4] OR631740
*Aspergillus sydowii*	CBS 593.65	MRCH [GenBank]	[28]	CBS	[aoxA] OR702890 [aoxB2-1] OR702889 [aoxB3] OR702891
*Trichoderma asperellum*	CBS 433.97	MBGH [GenBank]	[30]	CBS	[aoxA] OR683637 [aoxB2-2] OR683638

* cDNA was generated and sequenced as described in the Material and Methods section. cDNA sequences from ATG to stop codon were deposited at GenBank. ** *Penicillium rubens* strains were previously incorrectly called *P. chrysogenum*, a closely related but different species (recently reviewed by [31]). *** CBS: Centraal Bureau voor Schimmelcultures, currently known as the Westerdijk Fungal Biodiversity Institute (Utrecht, The Netherlands).

## Data Availability

Data are contained within the article and the associated Supplementary Materials. A list of the Aox sequences at the basis of the maximum likelihood phylogeny (Figure S1) can be provided by the Corresponding Author upon reasonable and formal (written) request. The determined *aoxA*, *aoxB1*, *aoxB2-1*, *aoxB2-2*, *aoxB3* and *aoxB4* sequences were deposited at GenBank under accession numbers OR702883, OR702884, OR683635, OR683636, OR702887, OR702888, OR702885, OR702886, OR714815, OR631741, OR631740, OR702890, OR702889, OR702891, OR683637, OR683638.

## Supplementary Materials

The following supporting information can be downloaded at https://www.mdpi.com/article/10.3390/jof9121195/s1, Figure S1: Maximum likelihood (ML) tree of 531 Aox proteins from 351 species of *Eurotiales*, *Onygenales* and *Lecanoromycetes* in circular format and the location of the four paralogous *aoxB* clades; Figure S2: evidence for a recent horizontal transfer of a series Flavi-born AoxB2-2 to the *Trichoderma asperellum* cryptic species complex; Table S1: oligonucleotide primers used to certify expression of alternative oxidase genes in seven species with multiple *aox* genes; Table S2: evidence of *aox* gene expression from RNA sequence read archives (SRA) that imply intron excision; Table S3: patterns of *aoxB* gene loss in *Aspergillus* sections and series.
