# Supplementary material for: Generation, Transfer, and Loss of Alternative Oxidase Paralogues in the Aspergillaceae Family
Source: J Fungi (Basel). 2023 Dec 14;9(12):1195. doi: 10.3390/jof9121195 (PMC10744626; doi:10.3390/jof9121195)
Supplement: Supplementary file 1 [file jof-09-01195-s001.zip › jof-2756459-supplementary.pdf]

Supplementary Materials (online)

Generation, Transfer, and Loss of Alternative Oxidase Paralogues in the *Aspergillaceae* Family

Michel Flipphi [1,†], Alexandra Márton [1,2,†], Vivien Bíró [1,2], Norbert Ág [1], Erzsébet Sándor [3], Erzsébet Fekete [1,*] and Levente Karaffa [1]

[1] Department of Biochemical Engineering, Faculty of Science and Technology, University of Debrecen, H-4032 Debrecen, Hungary; drir.michelflipphi@gmail.com (M.F.); martonszandi7@gmail.com (A.M.); biro0717@gmail.com (V.B.); agnorbi@gmail.com (N.Á.); levente.karaffa@science.unideb.hu (L.K.)
[2] Juhász-Nagy Pál Doctoral School of Biology and Environmental Sciences, University of Debrecen, H-4032 Debrecen, Hungary
[3] Institute of Food Science, Faculty of Agricultural and Food Science and Environmental Management, University of Debrecen, H-4032 Debrecen, Hungary; karaffa@agr.unideb.hu
[*] Correspondence: kicsizsoka@yahoo.com
[†] These authors contributed equally to this work.

Figure S1: Maximum likelihood (ML) tree of 531 Aox proteins from 351 species of *Eurotiales*, *Onygenales* and *Lecanoromycetes* in circular format and the location of the four paralogous *aoxB* clades.
Figure S2: Evidence for a recent horizontal transfer of a series *Flavi*-born AoxB2-2 to the *Trichoderma asperellum* cryptic species complex.
Table S1: Oligonucleotide primers used to certify expression of alternative oxidase genes in seven species with multiple *aox* genes.
Table S2: Evidence for *aox* gene expression from RNA sequence read archives (SRA) that imply intron excision.
Table S3: Patterns of *aoxB* gene loss in *Aspergillus* sections and series.

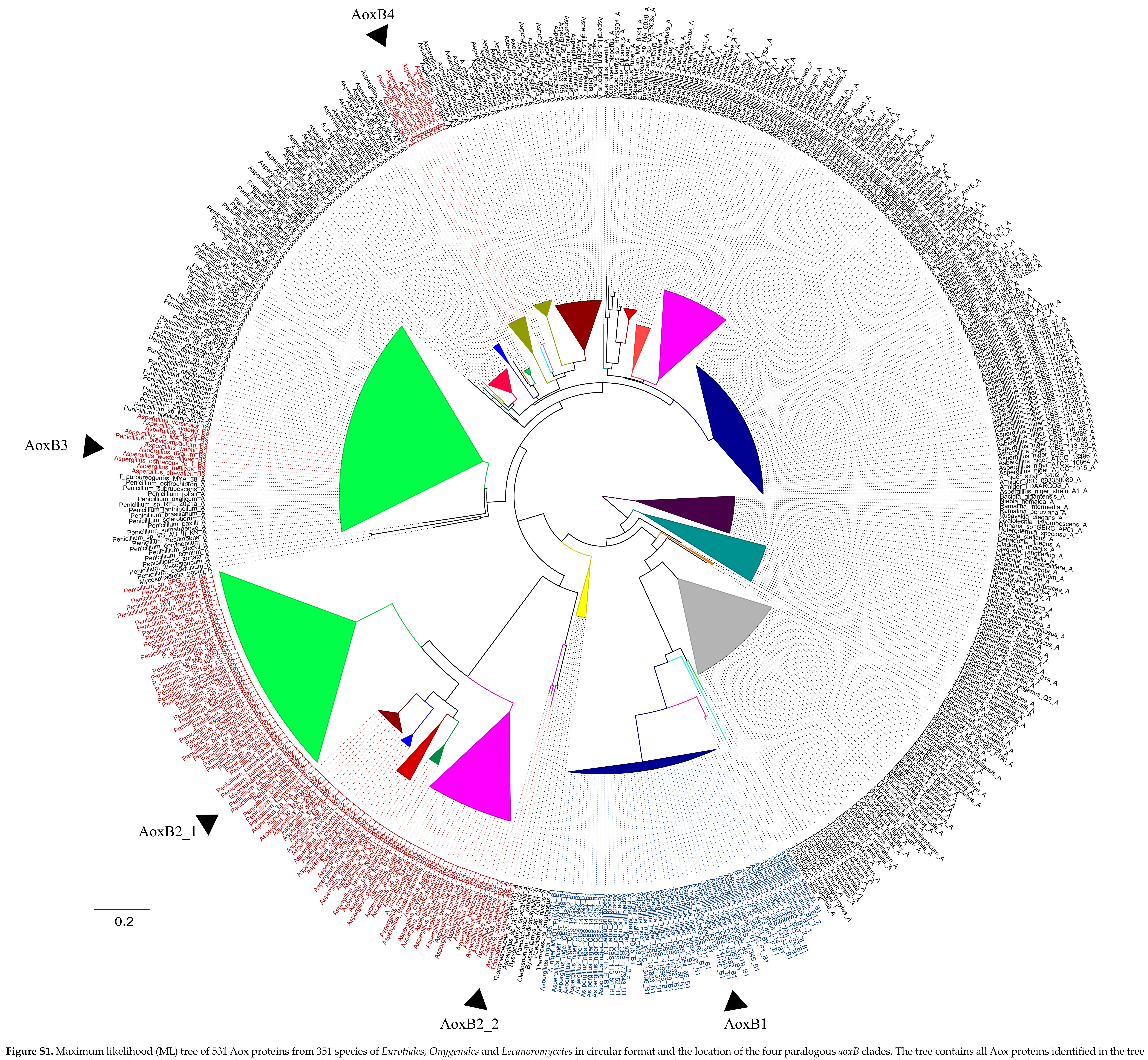

Figure S1. Maximum likelihood (ML) tree of 531 Aox proteins from 351 species of *Eurotiales, Onygenales* and *Lecanoromycetes* in circular format and the location of the four paralogous *aoxB* clades. The tree contains all Aox proteins identified in the tree taxa: most species have only the ubiquitous AoxA but some species of *Aspergillus* or *Penicillium* feature one or two additional full-length AoxB paralogues. Various taxonomic groups are cartooned for convenience. The clade of 24 *Lecanoromycetes* AoxA (dark purple cartoon) serves as the root of the phylogeny. The cartoons are coloured differently for the following taxa: blue green: *Trichocomaceae* family; orange: *Elaphomycetaceae* family; light grey: *Onygenales* order; yellow: *Thermoascaceae* family; lighter green: *Penicillium* genus (two clades); red: section *Fumigati*; blue: section *Candidi* (two clades); darker green: section *Terrei* (two clades); violet: section *Ochraceorosei*; olive green: section *Usti* (two clades); auburn: section *Nidulantes* including series *Versicolores*; turquoise: *A. wentii* representing section *Cremei*; crimson: section *Aspergillus* (two clades); tomato red: section *Circumdati* (two clades); magenta: section *Flavi* (two clades); dark blue: section *Nigri* including the *Aspergillus niger sensu stricto* complex (two clades). In the perifery all 531 protein names have been printed. Black letters are used for AoxA proteins; red letters mean paralogous AoxB; blue lettered are the *Aspergillus niger sensu lato* taxa with intact AoxB1. Outside the perifery, the four clades of paralogous proteins are named (anti clockwise) AoxB1, AoxB2-1 or AoxB2-2 for the evanishing satellite clade, AoxB3 and AoxB4, for the reader's convenience. The scattered existence of the rare duplication AoxB1 in four diverged taxa of *Aspergillaceae* was previously described.

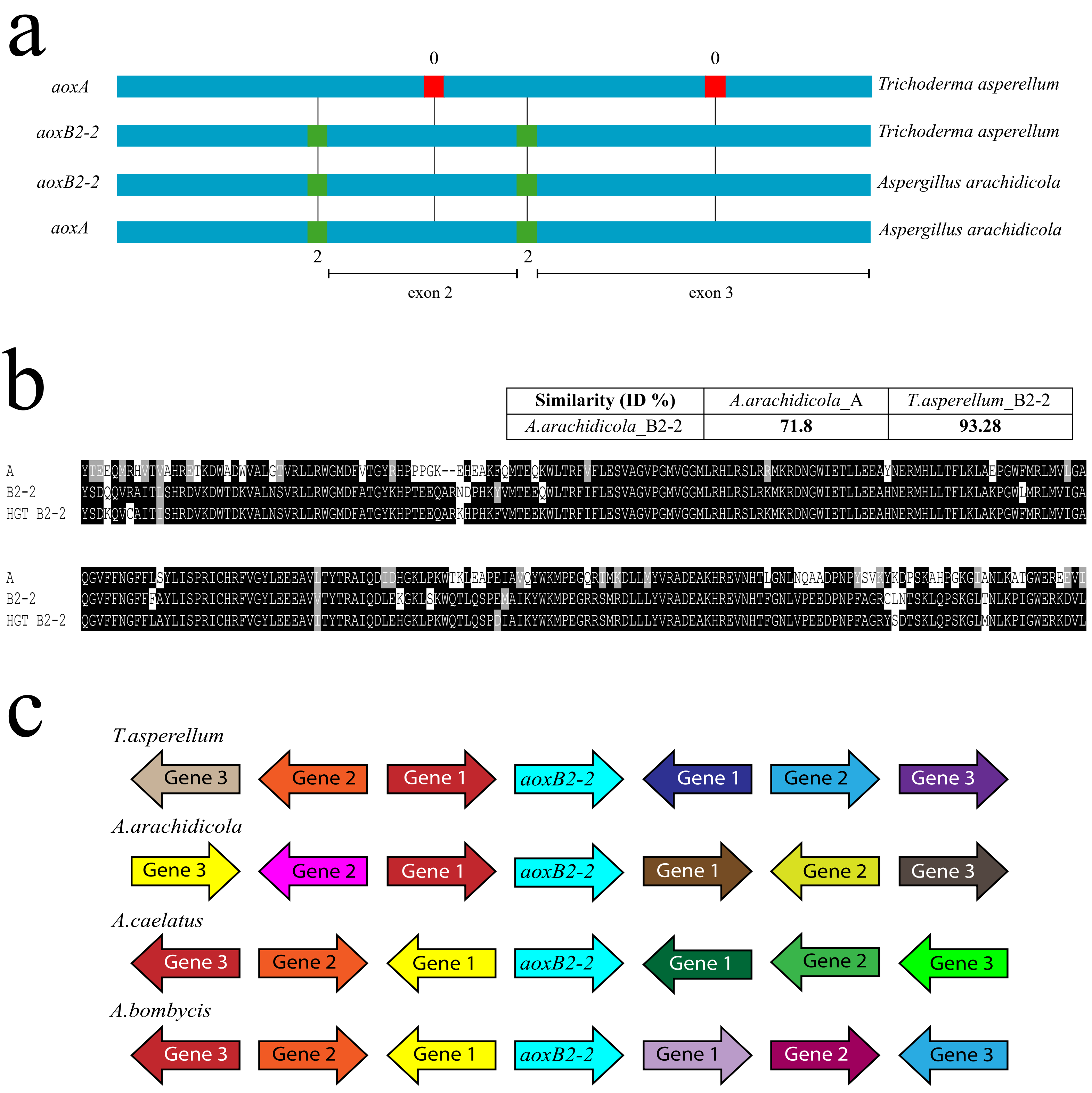

**Similarity (ID %)**	*A.arachidicola*_A	*T.asperellum*_B2-2
*A.arachidicola*_B2-2	**71.8**	**93.28**

Figure S2. Evidence for a recent horizontal transfer of a series *Flavi*-born AoxB2-2 to the *Trichoderma asperellum* cryptic species complex. (**a**) Schematic alignment of the gene models of the *A. arachidicola aoxA* and *aoxB2-2* with those of the *T. asperellum aoxA* and *aoxB2-2* genes. The transferred gene has the two phase-two introns (green) typical to *Eurotiales* and *Onygenales aox* genes. The *aoxA* gene from *T. asperellum* has two phase-zero introns (red) at completely different positions; (**b**) Triple sequence alignment of the C-terminal 266 amino acids of AoxA and the C-terminal 268 amino acids AoxB2-2 from *A. arachidicola* and AoxB2-2 from *T. asperellum*. The aligned peptides start with the conserved tyrosine [Y] encoded by first full codon in exon-2. Identical amino acids are shaded in black background; (**c**) The contemporary environment of the AoxB2-2 paralogous locus in *A. bombycis* (aka *A. luteovirescens*), *A. cealatus*, *A. arachidicola* and *T. asperellum*. The orientation and deduced function description of the product of the neighbouring genes were collected from the JGI genome browsers. The *aoxB2-2* paralogue gene in the centre is represented by the light grey arrows. The different colors represent different predicted functions for the neigbouring gene. For the sake of clarity, the differently colored arrows are all equal in size and thus do not represent the real size of the coding regions of the neigbouring genes.

Supplementary Table S1. Oligonucleotide primers used to certify expression of alternative oxidase genes in seven species with multiple *aox* genes.

Species	Primer	Sequence (5′-)
Aspergillus terreus NIH2624_A	Aterreus-aoxA-Fw	CCTCATTCAACGTTCATCGA
	Aterreus-aoxA-Rv	CTCAAAGGTACCTGGTCCCA
	Aterreus-aoxA-Bridge-Rv	CCAGTAGCGTTTCGATCCAT
Aspergillus terreus NIH2624_B2-1	Aterreus-aoxB2-1-Fw	CTAAAGAGTGGACCGCTCT
	Aterreus-aoxB2-1-Rv	ATGTAGGGCATTCACAGACA
	Aterreus-aoxB1-1-Bridge-Rv	GAGGAGGGTTTCGATCCA
Aspergillus oryzae RIB40_A	Aoryzae-aoxA-Fw	GTAGCTTCCCAATGTCCAGT
	Aoryzae-aoxA-Rv	CACAGTCCATAATGTGGATGAC
Aspergillus oryzae RIB40_B2-1	Aoryzae-aoxB2-1-Fw	GTACTCTTTCTCATCTGTCAG
	Aoryzae-aoxB2-1-Rv	AGAGGCAGAATTCTAGACCAA
	Aoryzae-aoxB2-1-Bridge-Rv	CCAGGAGTGTCTCAATCCA
Penicillium rubens NRRL1951_A	Prubens-aoxA-Fw	CTCTCTGTCATTGGTTCCATC
	Prubens-aoxA-Rv	GAGATGCTATTCAAATGACCAA
	Prubens-aoxA-Bridge-Rv	TCGAGCAGGGTTTCAATCCA
Penicillium rubens NRRL1951_B2-1	Prubens-aoxB2-1-Fw	CCTTGTGCAAGTCTCAAGAC
	Prubens-aoxB2-1-Rv	GATAGACCAGTTGAGGTCAA
	Prubens-aoxB2-1-Bridge-Rv	TCGAGGAGGGTTTCTATCCAT
Aspergillus wentii DTO 134E9_A	Awentii-aoxA-Fw	TCATCCATACGCATCAACCA
	Awentii-aoxA-Rv	CACGACGACAATGAGAAGGA
	Awentii-aoxA-Bridge-Rv	CTTCCAAAAGGGTTTCGATCCA
Aspergillus wentii DTO 134E9_B3	Awentii-aoxB3-Fw	CAGTATCAACTATTAAGCGACTA
	Awentii-aoxB3-Rv	CTGACATATATCCGTGGTCG
	Awentii-aoxB3-Bridge-Rv	CTAACTGTGTTTCAATCCAGC
Aspergillus calidoustus SF006504_A	Acalido-aoxA-Fw	CAGTACAACACTACGATCCA
	Acalido-aoxA-Rv	CCATAGCCTTTATCGTGTCA
	Acalido-aoxA-Bridge-Rv	CCAACAACGTTTCAATCCA
Aspergillus calidoustus SF006504_B1	Acalido-aoxB1-Fw	TGTTTGTGCTAGCAACTTCCA
	Acalido-aoxB1-Rv	GGTAACTCCATATGAAAGTCC
	Acalido-aoxB1-Bridge-Rv	CAATCAGCGATTCAATCCAT
Aspergillus calidoustus SF006504_B4	Acalido-aoxB4-Fw	CTTCTCGAATTCGGTCCAT
	Acalido-aoxB4-Rv	TGTACACAACACCTCCAAC
	Acalido-aoxB4-Bridge-Rv	CCAAAAGCGTTTCGATCCA
Aspergillus sydowii CBS 593.65_A	Asydowii-aoxA-Fw	CGATACATCCAATATCCAACCT
	Asydowii-aoxA-Rv	CGTCTTGACCAGTGAATG
	Asydowii-aoxA-Bridge-Rv	TCCAGCAAGGTCTCGATCCAT
Aspergillus sydowii CBS 593.65_B2-1	Asydowii-aoxB2-1-Fw	GGTACAACCTCACAGTCAAG
	Asydowii-aoxB2-1-Rv	CAGGTCATCAAGCGTCTAC
	Asydowii-aoxB2-1-Bridge	CAAGTAGTGTTTCAATCCA
Aspergillus sydowii CBS 593.65_B3	Asydowii-aoxB3-Fw	CGTTACTTGTTTGTATAGACATC
	Asydowii-aoxB3-Rv	CTCAAGAAATAGAACTATCGCAA
	Asydowii-aoxB3-Bridge	CGAGGAGCGTTTCTATCCA
Trichoderma asperellum CBS 433.97_A	Tasperellum-aoxA-Fw	CCACAGCTATATTTAGCTTTCAC
	Tasperellum-aoxA-Rv	ACACAACTTCGTTTCTTGAGTCT
Trichoderma asperellum CBS 433.97_B2-2	Tasperellum-aoxB2-2-Fw	GGATTGACATTAATAGCCAG
	Tasperellum-aoxB2-2-Rv	CACCTTGATAAAACGGTACA
	Tasperellum-aoxB2-2-Bridge	CTAGCAGTGTCTCGATCCA

Supplementary Table S2. Evidence for *aox* gene expression from RNA sequence read archives (SRA) that imply intron excision.

species / gene / intron no.	exon fusion	NCBI SRA database	SRA read covering exon fusion
A. calidoustus			
aoxA intron 1	CCAGTTTAT	ERX631988	ERR677097.21482935.1
intron 2	GGATGGATT	ERX631988	ERR677097.21338014.1
intron 3	CCTTCCTCAA	ERX631988	ERR677097.21534348.1
aoxB1 intron 1	CCAATCTAC	ERX631988	ERR677097.19174082.1
intron 2	GGATGGATT	ERX631988	ERR677097.383320.1
aoxB4 intron 1	CCGGTGTAC	ERX631988	ERR677097.4402296.2
intron 2	GGCTGGATC	ERX631988	ERR677097.11324119.1
A. oryzae			
aoxA* intron 1	CCTGTTTAT	DRX169184	DRR178658.23709178.1
aoxB2-1 intron 1	CCTGTATAC	DRX169184	DRR178658.3184879.2
intron 2	GGATGGATT	DRX169184	DRR178658.21178077.1
A. sydowii			
aoxA intron 1	CCTGTCTAT	SRX13745574	SRR17576387.22532111.1
intron 2	GGATGGATC	SRX13745574	SRR17576387.22175180.1
aoxB2-1 intron 1	CCAGTATAC	SRX13745574	SRR17576387.21790899.1
intron 2	GGATGGATT	SRX13745574	SRR17576387.19454217.1
aoxB3 intron 1	CCAGTATAC	SRX13745574	SRR17576387.22181706.1
intron 2	GGGTGGATA	SRX13745574	SRR17576387.22515080.1

Species / gene / intron no.	exon fusion	NCBI SRA database	SRA read covering exon fusion
A. terreus			
aoxA intron 1	CCTGTCTAT	SRX7896641	SRR11291067.6645117.1
intron 2	GGATGGATC	SRX7896641	SRR11291067.4382190.1
aoxB2-1 intron 1	CCAGTGTAC	SRX7896641	SRR11291067.4577324.1
intron 2	GGCTGGATC	SRX7896641	SRR11291067.6484527.1
A. wentii			
aoxA intron 1	CCTGTTTAC	Aspwe1 [JGI]	No excision detected Locus2300v1rpkm79.34
intron 2	GGATGGATC	Aspwe1 [JGI]	Locus5976v1rpkm17.33 [EST contig]
aoxB2-1 intron 1	CCAGTATAC	Aspwe1 [JGI]	Locus8961v1rpkm3.92 [EST contig]
intron 2	GGCTGGATT	Aspwe1 [JGI]	Locus8961v1rpkm3.92 [EST contig]
P. rubens			
aoxA intron 1	CCAGTATAC	SRX7124595	SRR10428551.21351875.1
intron 2	GGATGGATT	SRX7124595	SRR10428551.1426888.1
aoxB2-1 intron 1	CCCGTATAC	SRX7124595	SRR10428551.4681329.1
intron 2	GGATGGATA	SRX7124595	SRR10428551.20234005.1
T. asperellum			
aoxA intron 1	AATGGCTTGT	SRX19144862	SRR23195941.7227219.2
intron 2	TTCAATACTG	SRX19144862	SRR23195941.2092837.1
aoxB2-2 intron 1	CCGGTGTAC	SRX19144862	SRR23195941.7589312.2
intron 2	GGTTGGATC	SRX19144862	SRR23195941.13228317.1

Footnote: Extant NCBI-lodged RNA SRAs are recorded that cover the predicted exon-exon fusions in alternative oxidase transcripts in the seven species listed in Table 1. Only one SRA is recorded for each intron excision. For *Aspergillus wentii* there are no RNA SRAs; instead, appropriate EST contigs from JGI were collected and listed. * The second intron in *aoxA* (ubiquitous gene) is absent from all genome-sequenced section *Flavi* species.

Supplementary Table S3. Patterns of *aoxB* gene loss in *Aspergillus* sections and series.

Species	Section	Series	*aoxA*	*aoxB1*	*aoxB2-1*	*aoxB2-2*	*aoxB3*	*aoxB4*
***A. bombycis* (*A. luteovirescens*)**	*Flavi*	*Nomiarum*	X		X	X		
A. nomiae		*Nomiarum*	X		X			
A. caelatus		*Kitamyces*	X		X	X		
A. tamarii		*Kitamyces*	X		X			
A. arachidicola		*Flavi*	X		X	X		
A. parasiticus		*Flavi*	X		X			
A. flavus		*Flavi*	X		X			
A. leporis		*Leporum*	X		X			
A. hancockii		*Leporum*	X		X			
A. alliaceus		*Alliacei*	X		X			
A. bertholletiae		*Bertholletiarum*	X		X			
A. calidoustus	*Usti*	*Calidousti*	X	X				X
A. pseudodeflectus			X					X
A. germanicus			X					X
A. keveii			X					X
A. insuetus			X					X
A. carlsbadensis			X					X
A. ustus		*Usti*	X					X
A. chevalieri	*Aspergillus*	*Chevalierorum*	X		X		X	
A. montevidensis			X		X			
A. intermedius			X		X			
A. caperatus			X		X			
A. cibarius		*Aspergillus*	X		X			
A. glaucus		*Aspergillus*	X					
A. cumulatus		*Rubri*	X		X		X	
A. ruber		*Rubri*	X					
A. xerophilus		*Xerophili*	X		X			
A. punctus	NA	NA	X		X			
A. penicilloides	*Restricti*	*Penicillioides*	X		X			
A. implicatus	*Sparsi*	*Implicati*	X	XX				
***Aspergillus sp.* MA 6041**	NA	NA	X		X		X	
A. sydowii	*Nidulantes*	*Versicolores*	X		X		X	
A. creber			X		X			
A. teennsseensis			X		X			
A. jensenii			X		X			
A. austroafricanus			X		X			
A. amoeneus			X		X			
A. protuberus			X		X			
A. versicolor			X		X			

A. subversicolor			X		X			
A. angustatus		*Stellati*	X		X		X	
A. wentii			X				X	
A. sepultus		*Wentiorum*	X				X	
A. dimorphicus	*Cremei*		X				X	
A. arxii		*Arxiorum*	X				X	
A. inflatus		*Inflati*	X				X	
A. insolitus	*Polypaecilum*	*Polypaecilum*	X		X			
A. hortai			X		X			
A. pseudoterreus			X		X			
A. terreus		*Terrei*	X		X			
A. alabamensis	*Terrei*		X		X			
A. floccosus			X		X			
A. auroterreus			X		X			
A. ambiguus		*Ambigui*	X		X			
A. candidus			X		X			
A. campestris			X		X			
A. taichungensis	*Candidi*	*Candidi*	X		X			
A. subalbidus			X		X			
A. triticus (A. tritici)			X		X			
A. westerdijkiae			X				X	
A. ochraceus			X				X	
A. melleus			X				X	
A. ostianus	*Circumdati*	*Circumdati*	X				X	
A. sesamicola			X				X	
A. westlandensis			X				X	
A. subauricomus			X				X	
A. petrakii	NA	NA	X				X	
A. neoaricomus	NA	NA	X				X	
A. uvarum	*Nigri*	*Japonici*	X		X			
P. brevicompactum	*Brevicompacta*	*Brevicompacta*	X		X		X	
P. brasilianum	*Lavata-Divaricata*	*Simplicissima*	X		X			X
P. swiecickii	*Ramosum*	*Lanosa*	X	X	X			

*NA. Fungal isolate not analyzed and categorized by Houbraken and co-workers [32].

Footnote: Species from the *Aspergillus* whole-genus sequencing project (JGI Proposal ID: 1307; written with red letters in the left column) were assessed on the basis of their assignment to sections and/or series – according to Houbraken and co-workers – in which species with two or three *aox* genes (written with black letters in the left column) were earlier identified. In the six columns at the right, the presence of *aox* genes was indicated by the crosses [X]: for convenience, these indicators for the independent *aox* duplication events are in different colors – black (*aoxA*), turquoise (*B1*), green (*B2-1*), violet (*B2-2*), yellow (*B3*) and magenta (*B4*), respectively – and further highlighted by the yellow- or black background.
